# Tetanus

**Published:** 1887-12

**Authors:** L. L. McArthur


### THE MEDICAL JOURNAL AND EXAMINER.

VOL. LV. DECEMBER, 1887. No. 6.

### TETANUS.

BY L. L. McARTHUR, M. D.

READ BEFORE THE CHICAGO MEDICAL SOCIETY, ON DECEMBER 5TH.

In presenting the following paper to this society, the intention has been rather to provoke renewed discussion upon an important subject already oft gone over, than with the hope of presenting any very original work. If by so doing new ideas with new experiences are added to our meager fund of information, concerning the causation and treatment of this rare and fatal disease, as well as the clearing away of any inherited cobwebs bequeathed by our medical ancestors, the work will not have been in vain.

Before entering upon the discussion of the subject proper, let me state that those diseases to which the term tetanus is applied, as a complication or phase, such as puerperal tetanus, tetanus neonatorum, tetanilla, etc., will not be included under the caption of this paper, inasmuch as more or less satisfactory explanations of their etiology, pathology and treatment are extant.

It is defined as "a disease characterized by permanent and painful contractions of the voluntary muscles, wholly or in part, habitually beginning in those of the neck and inferior maxilla, and extending thence to others."

Nicaise makes the best subdivision of the disease, viz:

1 Acute.

2 Sub-acute.

3 Chronic.

While others, intending to hint at the etiology, add to the above—

Tetanus e frigore (from cold).

Tetanus traumaticum.

Tetanus spontaneous, further modifying these by adding special terms for the special groups of muscles involved, as *trismus* for the jaw, *opisthotonos*, for the extensors of the trunk, *emprosthotonos*, for the flexors of the trunk, or for the special character of the contractions which may be (1) continuous, (2) intermittent.

### ETIOLOGY.

When more unanimity exists as to the causation of this disease more rational therapeutics as well as satisfactory results can be expected; and I trust that a reflection of the opinions to-day current with recognized authorities may indicate a step in advance to us through the labyrinth of suggested causes.

This much is so well known as to be *almost* axiomatic, that every case of tetanus has, *as a primary factor*, a traumatism, be that traumatism so small as the prick of an hypodermic needle or so extensive as the crushing of a limb, so slight as a burn upon the forearm, or so extensive as the contusion *without laceration* of the buttocks incident to a fall from a horse's back. Is it strange, then, that the primary traumatism is often overlooked? Would not many a "*spontaneous* tetanus" change its name to "traumatic," had the physician had emphasized for him the fact that in this disease, as in hydrophobia, to which it bears a strong analogy, the primary, and, may I say infecting, wound has often long since healed ere the dreaded symptoms make their appearance.

*Race* proclivities are mentioned as a factor predisposing the blacks to more frequent attacks than whites; while the countless other causes advanced, if considered in the light of the recent progress in bacteriology, serve but to explain the introduction of the materies morbi, virus, germ—call it what you will—which constitutes the *second factor* in the etiology of tetanus.

Then becomes clear the predisposition which *occupation*, as hostler, stable-boy and gardener induces, since these are affected more frequently than any others.

*Climates.* Where sudden alternations between heat and cold, with moisture, occur, thus favoring the growth of vegetable matter, the disease is more frequent, even in epidemics among the lower animals, as often occurs in India. The depressing influence of *wet and cold* on the wounded was clearly shown upon the battle-field of Botzen. Verneuil claims, and the mass of evidence now collected certainly tends that way, that tetanus is *always* an infectious, never a spontaneous, malady — sustaining his claims by pointing out the frequency with which hostlers, who deal with the animal most often attacked, acquire the disease; the frequency with which the history of tetanus in the groom is preceded by the same in his horse. This is sustained in two ways: First—*Clinically.* Well-authenticated cases distinctly traceable to wounds received while treating tetanic horses, the case of the late Dr. Wing, a veterinary surgeon of New York, being one in point. While making a post-mortem on the cadaver of a horse which had died of tetanus, he accidently scratched his hand, acquired the disease and died. Secondly—*Experimentally.* By the investigations of Rosenbach, who, while studying the hygienic influences of garden soil, eliminated and cultivated therefrom a microörganism which, when introduced beneath the skin of the lower animals — except the dog, which is exempt therefrom—invariably induced tetanus, even through any number of cultures. Small masses of muscular tissue, carefully excised from a case of tetanus and introduced into the subcutaneous tissue of lower animals, promptly induced the characteristic phenomena.

Clinically, again, this receives great support when one considers the disproportion of tetanus occurring in wounds of upper and lower extremities, as reported in the Surgical History of our late war.

In 16,000 foot-injuries 57 cases of tetanus ensued.

In 12,000 hand-injuries not a single case was reported.

Here the possibility for wound-infection of the foot was much greater from the soil than in the case of the hand. If further evidence were wanting to prove that there must be a specific element, it may be supplied by the cases, reported by Betoli, of three slaves, who, although specially cautioned not to eat of the flesh of a horse dead of tetanus, did eat of it, and all contracted the disease, and all died of it.

To test the truth of Rosenbach's statements, both Flügge and Nicolaies repeated his experiments with positive results.

Then Socin, of Basle, made the direct test by introducing garden soil subcutaneously, with the result of inducing tetanus.

Before leaving the subject of etiology mention ought to be made of the theory of E. Rose, that tetanus is due to an ascending neuritis. This seems to be true as revealed by post-mortem examination in some cases; but what advance have we then made—for something must cause the inflammation?

This induces the reflection that just as the germ of hospital gangrene would spare the muscles, but dissect away their sheaths and connective tissue, so that of tetanus may have a predilection for nerve tissue, and thus explain the neuritis.

Gautier, who has advanced toxicology so much by his investigations of cadaveric alkaloids, leucomaines and ptomaines, furnishes by far the most acceptable explanation of the phenomena of tetanus-poisoning, by having extracted from the fluids in which various germs have grown alkaloidal bodies of fixed chemical and physiological properties, but differing for each microörganism. Thus the micrococcus of septicæmia invariably produces an alkaloid named septicine of definite chemical properties, which can be extracted by the usual methods for obtaining alkaloids, and which, when introduced into the circulation, produces temporarily all the phenomena of septicæmia, ephemeral fever; so, too, from the culture-fluid of bacillus tuberculosis an alkaloid can be obtained producing temporary hectic, followed by by sweating, etc. One of these alkaloids, extracted by Gautier reacts upon the organism like the strychnia group, and induces tetanic convulsions identical with those of tetanus.

DIAGNOSIS.

In regard to the differential diagnosis between tetanus and allied diseases, some points will bear reference here, though to cover the entire ground might seem a work of supererorgation.

Reëmphasizing Rosenthal's remark, "*Many cases* have been reported as tetanus which were merely *varieties* of *tonic spasms*, affecting the trunk and limbs. In all these the characteristic sign of morbid increase of reflex action has been lost sight of," I would say that special care should be used to differentiate this disease from hydrophobia, hysterical tetanus, the masticatory spasm of Romberg, and certain tonic spasms resulting from obscure meningeal inflammations of the base of the brain. In *hydrophobia* the history, combined with the usual six weeks' period of incubation, whilst that of tetanus generally being within twelve days, will usually suffice to differentiate; but without these, cases so closely simulate it that, as before mentioned, the French school have made a special class for such "tetanus hydrophobica." Some of the early-developing, alleged, cases of hydrophobia may have been simple traumatic tetanus, the history of a bite having misled the diagnostician. In hydrophobia there is usually periodic unconsciousness, alternating with delirium, while in tetanus the mind remains clear to the last.

*Hystero-tetanus* of a marked character can usually be differentiated by the thermometer; it is of a quick and benign course.

Romberg has called attention to a spasm of the masticators supplied by motor branches of the trigeminus as a result of cerebral softening, cerebritis, and circumscribed meningitis. This, if superficially observed, might be considered as a localized tetanus, called trismus. Tetanic conditions, it is to be remembered, complicate many acute diseases; but their individual peculiarities here point to the true diagnosis.*

*The acute* form might justly be called tetanus fulminans, so sudden and severe is its onset and course. Three days of high temperature, severe convulsions, terminating always fatally at the end of the fourth day with spasm of the larynx, or heart-failure, possibly from remedial agents employed. Spasms yield only to chloroform or ether.

In *the subacute* form, the symptoms are slower in appearing and not so severe in their character, with fifty per centum of recoveries, having an average duration of twelve days, while in the *chronic form* we have no abnormal temperature, the spasms are slight, and terminate favorably in thirty to sixty days. In fact, the fatality of tetanus is in direct ratio to the acuteness of the attack, as Dr. Moses Gunn has stated.

Further subdivisions have recently been added to the usual classes, of which *Kopftetanus* of the Germans is applied to a tetanic spasm of the muscles of the jaw and face, in which the spasm of the muscles on one side the face being greater than on opposite side, causes the same distortion that accompanies facial paralysis. This variety is very insidious in its onset as well as always fatal.

Again, we have the tetanus hydrophobica of the French school; in that variety the spasms affect the muscles of the pharynx and larynx largely. It is of the greatest importance to the patient that the diagnosis be made early, because incipient cases yield more readily than than those well advanced.

As the knowledge of nervous diseases becomes better, I venture to predict that two or even three diseases possessing many of the characteristics of tetanus and each other, will be differentiated from the general class tetanus into sub-classes of their own, and found to be due to other causes.

DIFFERENTIAL DIAGNOSIS.

Three to five per centum of all cases are of the partial character.

In lieu of the clinical history and course of the the disease, let me give briefly the history of one of my cases:

James Cash, aged 31, a switchman by occupation, of good physique and general health, was brought to St. Luke's Hospital for a crushing injury of the foot, received on Illinois Central railroad, on September 26, 1887. While coupling cars in motion and walking backward between them, his foot caught in a frog. With difficulty he pulled off his boot and threw himself to one side, but received a crushing injury by a flange of a wheel which ran obliquely across the heel, and crushed the posterior half of the os calcis. On reaching the hospital the wound, which was full of dirt, was washed, irrigated and dressed antiseptically. During the night he had a slight hæmorrhage, for which a bandage was applied. He admitted having been drinking; otherwise his general conditions had always been excellent. There was no history of any nervous diseases in his family.

Careful microscopical examination was made of numerous sections of the sciatic,

*Due acknowledgments are here made to Nicaise's article in Ashurst "System of Surgery;" *Medical Record, Progres Medicale and Wiener Med. Wochenschrift.*

removed under antiseptic precautions, but nothing could be found there of a bacterial character. A post-mortem examination not being permitted, it was impossible to obtain the tissues situate near the wound; so the result, while negative, was not of much weight. Punctate hæmorrhages, with infiltration of the connective tissue sheaths by leucocytes, were noticed.

Nothing of special interest occurred from September 26th, the date of his admission, outside the usual surgical dressing, which had been more or less painful, and the temperature ranging between 100° F. 103.5° for which quinine was given. Nothing more occurred than was to be expected with the wound, until October 9th, when the bowels, having been somewhat constipated —a prodromal symptom—a cathartic of ol. Ricini was ordered at night for him. October 10th, at 4 P. M., he began to complain of pain between his shoulders, and his hands and arms were twitching slightly. Ordered Bromidia ʒss. every three hours, p. r. n. His diet was milk, egg-nogg, light food. 11th, A.M. Slept little during the night. The pain between the shoulders continued, and his face was drawn at the angles of his mouth. P. M. Slight opisthatonos, and I increased the Brom. to ʒi. every three hours. October 12th Risus Sardonicus was marked, but says he feels good; look of contentment on face; trismus requires stick between *teeth* to avoid gritting *them;* says he is *much better*, Bromide ʒss. *every two hours.* 13th; some difficulty in swallowing showing itself. Dress foot myself. Give ether, and open up the entire sole of foot. Wash out with 95 per cent. Found sphacelus of connective tissue. Bromide ʒi, chloral gr. 10, every two hours. 14th, worse; every symptom worse; chloroform during the night; stretch sciatic; Physostigmatis, gr. ⅛ every hour.

15th, spasms were quieted by operations; they returned at 6 P. M., and during night were very bad. Chloroform 3 times during night. Respiration greatly interferred with. Death on 16th, A. M. No post-mortem permitted.

*Treatment.*—As the result of my investigations of the history of the cases collected between 1882 and 1887, nervous sedatives seem to yield the best results when combined with the most absolute quiet and rest. Gross says in forty years' practice he had seen but three cases recover—then only after a protracted struggle, bromides and opium being the main-stay.

| PHYSICIAN. | CASES. | RECV'Y | DEATH. | TREATMENT. |
|---|---|---|---|---|
| Cheesman | 1 | 1 | | Whisky to intoxication. |
| Acentz | 1 | 1 | | Venesection, Morph. 5 cent, hypoderm. rec. |
| Alderson | 3 | 3 | | Bromide and chlor, aa., 25 grs., 2 hours. |
| Gunn | 3 | 2 | 1 | Physostigma Nerve stretching. |
| Stair | 2 | 1 | 1 | Heroic chloral and bromide. |
| Verneuil | 7 | 5 | 2 | Chloral & brom. Quiet and rest. 1 sudden death. |
| Jackman | 1 | 1 | | Urethran; after failure, chloral. |
| Mayer | 1 | 1 | | Chloral and bromide. |
| Brown | 1 | 1 | | Quin., enormous doses; 100 grains every hour. |
| Beck | 36 | 21 | 15 | Chloral. |
| Wood | 13 | 11 | 2 | Br. Pot. |
| Kolipinski | 1 | 1 | | Recovery; chloral. |
| R. H. McCord | 3 | 2 | 1 | Chloral. |
| McArthur | 4 | 3 | 1 | Nerve stretching in 1. Calabar bean in other. |
| Total | 77 | 54 | 23 | 1 Imbecile. |

A glance at the foregoing table will show that as yet our main reliance must be on the symptomatic treatment, using as nervous sedatives those best known to us as such. The most absolute quiet is essential, with protection from all external sources of irritation, such as noises or sudden draughts.

